# Locally Controlled Release of Methotrexate and Alendronate by Thermo-Sensitive Hydrogels for Synergistic Inhibition of Osteosarcoma Progression

**DOI:** 10.3389/fphar.2020.00573

**Published:** 2020-05-19

**Authors:** Hongli Shan, Ke Li, Duoyi Zhao, Changliang Chi, Qinyuan Tan, Xiaoqing Wang, Jinhai Yu, Meihua Piao

**Affiliations:** ^1^Department of Clinical Laboratory, The First Hospital of Jilin University, Changchun, China; ^2^Department of Orthopedics, the Fourth Affiliated Hospital of China Medical University, Shenyang, China; ^3^Department of Urology, the First Hospital of Jilin University, Changchun, China; ^4^Department of Gastrointestinal Surgery, The First Hospital of Jilin University, Changchun, China; ^5^Department of Anesthesiology, The First Hospital of Jilin University, Changchun, China

**Keywords:** thermo-sensitive hydrogel, local injection, controlled drug release, osteosarcoma, synergistic chemotherapy

## Abstract

Osteosarcoma (OS) is a serious primary bone malignant tumor that can easily affect children and adolescents. Chemotherapy is one of the important and feasible clinical treatment strategies for the treatment of OS at present, which is severely limited due to insufficient retention time, poor penetration ability, and serious side effects of current anti-tumor drug preparations. In this work, a novel injectable thermo-sensitive hydrogel (mPEG_45_–PLV_19_) loaded with methotrexate and alendronate, and the sustained release at the tumor site synergistically inhibited the progression of OS. The mPEG_45_–PLV_19_ shows excellent physical and chemical properties. Compared with other treatment groups, the *in vivo* treatment of gel+ methotrexate + alendronate effectively inhibited the growth of tumor. More importantly, it significantly reduced bone destruction and lung metastasis caused by OS. Therefore, this injectable thermo-sensitive hydrogel drug delivery system has broad prospects for OS chemotherapy.

## Introduction

Osteosarcoma (OS) is a malignant bone tumor that occurs mainly in the proximal tibia and distal femur in children and adolescents (Kosei et al., 2013; Nedelcu et al., 2014; Xu et al., 2019). Even with adequate clinical treatment, 5-year overall survival for OS patients is still less than 60% (Ottaviani and Jaffe, 2009; Zhang et al., 2018a; Zhang et al., 2018b). And when OS patients have metastasis or recurrence, their 5-year survival rate drops to less than 20% (Wu et al., 2009).

Current clinical treatment for patients with OS includes preoperative chemotherapy, intraoperative resection of the lesion (including metastasis), and postoperative chemotherapy (Isakoff et al., 2015; Li et al., 2018). Systemic chemotherapy is currently one of the most important means of clinically suppressing the progression of OS. However, systemic chemotherapy is often accompanied by the following severe problems: (1) poor selectivity of chemotherapy drugs (Chen et al., 2017; Gao et al., 2019; Sun et al., 2019; Ma et al., 2020); (2) strong side effects (Feng et al., 2019a; Feng et al., 2019b; Wang et al., 2019); (3) drug resistance (Ding et al., 2019a; Ding et al., 2019b; Ding et al., 2019c); (4) tumor recurrence, etc (Kim and Helman, 2009; Marco and Sch?Fer, 2010; Moorthi et al., 2011). Therefore, there is an urgent need for new and effective therapy for treating OS.

Hydrogels are materials of a 3D network structure formed by physical or chemical crosslinking (Du et al., 2019). Injectable hydrogels are now common in biomedical applications (Zhang W. et al., 2018). Among them, temperature-responsive hydrogels are particularly noteworthy (Du et al., 2019; Mu et al., 2019). Thermo-sensitive hydrogel is a special kind of injectable biomaterials (Huang et al., 2019; Zarrintaj et al., 2019). The solution is in a sol state and has fluidity at low temperatures, which is extremely beneficial for drug loading (Ta et al., 2008; Wang et al., 2015). The mixed solution is injected into the tumor site, and the solution rapidly changes from the sol state to the gel state upon stimulation of body temperature. Then, the hydrogel slowly degrades in the body due to the interaction between its own molecules, causing its loaded drug to slowly release from the hydrogel (Seeli and Prabaharan, 2017). Localized treatment of thermo-sensitive hydrogel has been reported to include the following advantages: (1) sustained release of drug at the tumor site; (2) reduced administration time and systemic side effects; (3) overcoming the low solubility of the chemotherapeutic drug; (4) small surgical trauma and simple operation; (5) patients’ compliance and comfort level were improved (Lovett et al., 2015; Xiong et al., 2015; Wu et al., 2016).

Therefore, based on the above characteristics, a temperature-sensitive poly(L-valine) (PLV) hydrogel was developed in this work. First, a ring-opening polymerization (ROP) of L-valine N-carboxy anhydride (L-Val NCA) is initiated by terminally aminated polyethylene glycol monomethyl ether (mPEG_45_-NH_2_) to form mPEG_45_–PLV_19_. Secondly, the chemical structure characteristics of temperature-sensitive PLV hydrogels were identified by proton nuclear magnetic resonance spectroscopy and Fourier transform infrared spectroscopy (FT-IR). Then, the change of the state of PLV hydrogel with temperature was analyzed by tube inversion method, and the mechanical properties were further analyzed by rheological analysis. The surface morphology of PLV hydrogel was obtained by scanning electron microscopy (SEM). Thereafter, the biodegradability of PLV hydrogel was analyzed by an *in vivo* environment simulated *in vitro*. Hematoxylin and eosin staining (H&E) were used to observe the biocompatibility of PLV hydrogels. Methotrexate (Mtx) is one of the first-line chemotherapy drugs for OS, which mainly inhibits tumor cell synthesis by inhibiting dihydrofolate reductase. Alendronate (Aln) is the most important anti-resorber for the treatment of bone diseases. Aln has a high affinity for bone minerals and prevents bone destruction by inhibiting osteoclast activity. Finally, the PLV hydrogel was loaded with Mtx and alendronate Aln to inhibit orthotopic OS in mice. The Gel+Mtx+Aln group was observed to have the greatest tumor suppressive effect, with minimal lung metastasis and minimal bone destruction. In summary, the temperature-sensitive PLV hydrogel combined with Mtx and Aln as a novel drug delivery system has shown good application prospects in the local synergistic chemotherapy of OS.

## Materials and Methods

### Materials

Methoxy poly(ethylene glycol) (mPEG_45_, 98%) and *n*-hexylamine (99%) was purchased from Sigma-Aldrich (St. Louis, MO, USA). L-Valine (L-Val, 98%) is from GL Biochemicals Co., Ltd. (Shanghai, P. R. China). Mtx (99%) and alendronate (97%) was purchased from Sigma-Aldrich (St. Louis, MO, USA). Triphosgene (98%) was purchased from Shanghai Duodian Chemical Co., Ltd. (Shanghai, P. R. China). *p*-Toluenesulfonyl chloride (99%) was purchased from Sinopharm Chemical Reagent Co., Ltd (Shanghai, P. R. China). *n*-Hexane (99%), toluene (99%), ethyl acetate (99.8%), *N*,*N*-dimethylformamide (DMF, 99.5%), and diethyl ether (98%) were purchased at Beijing Chemical Plant (Beijing, P. R. China). Among them, *n*-hexylamine and DMF were purified by a solvent treatment system.

### Synthesis of mPEG_45_–PLV_19_

L-Val NCA ROP was initiated by using mPEG_45_-NH_2_ as a macroinitiator to obtain mPEG_45_–PLV_19_ block copolymer. Weigh a portion of mPEG_45_-NH_2_ 2.0 g and 150.0 ml of anhydrous toluene at 130°C for 2 h to azeotropically remove water. Subsequently, the toluene was gradually taken out, and the cold trap was evacuated for 1 h to remove the residual toluene in the reaction flask. Then 2.86 g (0.02 mol) of L-Val NCA was added and mixed with 100.0 ml of DMF. The polymerization was stirred magnetically at 25°C for 3 d, next settled with 1,000.0 ml of ice diethyl ether. The obtained product was dissolved in 40.0 ml of DMF and dialyzed (molecular weight cut-off [MWCO] = 3,500 Da) for 72 h. The dialyzed water was changed every 6 h. Finally, the final product, mPEG_45_-PLV_19_, was obtained by lyophilization.

### *In Vitro* Drug Release

The copolymer solution (500 ul, 5% wt%) was added to a 3.0 ml glass vial and incubated for 10 min at a constant temperature (37°C) to obtain an mPEG45-PLV19 hydrogel. Buffer solution (2.0 ml) was added to the top of the hydrogel at 37°C, and the medium was changed once a day. Determine the concentration of MTX or Aln released by a UV spectrophotometer.

### Establishment of an Orthotopic OS Model

K7M2 cells were collected and then mixed with PBS. The mice were anesthetized with ether and depilated to fully expose the surgical site. Using a 1 ml syringe needle, the cortical layer of the right tibia was pierced vertically and the needle was inserted into the metaphysis of the tibia for approximately 3.5 mm. After the needle was removed, 2 million K7M2 cells (30 ul) were slowly injected into the bone marrow using a micro-injector. Tumor growth in the right leg of mice was monitored daily, while the tumor volume (V) was measured by micrometers. Mice were euthanized when the experiment was over (16 d) or when the tumor size reached 1 cm^3^.

### *In Vivo* Anti-Tumor Efficacy

After inoculation of K7M2 tumors for 3–4 weeks, when the right tibia tumors of the mice were visible (volume about 200 mm^3^), the mice began to receive treatment. The Mtx and the Aln dose was 50 mg/kg. The gel (5.0 wt.%) containing Mtx and Aln was injected in the sol state (4) to inoculate 100 ul to the vicinity of the mouse *in situ* tumor. Tumors were measured at the top (AP) and longitudinal (L) using a digital caliper. AP was measured at the knee joint, and L was measured along the long axis of the tibia. Estimate the size of the primary tumor (V) according to formula (Zhang W. et al., 2018) (1):

(1) $$V({\text{mm}}_{3})=\frac{4\pi }{3}{\left(\frac{AP+L}{4}\right)}^{2}$$

Euthanasia was performed after all treatments were completed, and the hind limbs with tumors were collected and photographed.

### Micro-CT

The right tibia of the mouse was collected and placed on a micro-CT specimen table to evaluate the damage of the humerus bone by radiographic analysis. The X-ray tube voltage was set to 40 kV. The data is derived from a 360° individual projection collected every 1° of rotation, rotated one week around the specimen, and then reconstructed using computer software for 3D reconstruction (Recon; PINGSENG, Shanghai, China).

## Results and Discussion

### Fabrication and Characterization of mPEG_45_-PLV_19_

**Figure 1:**
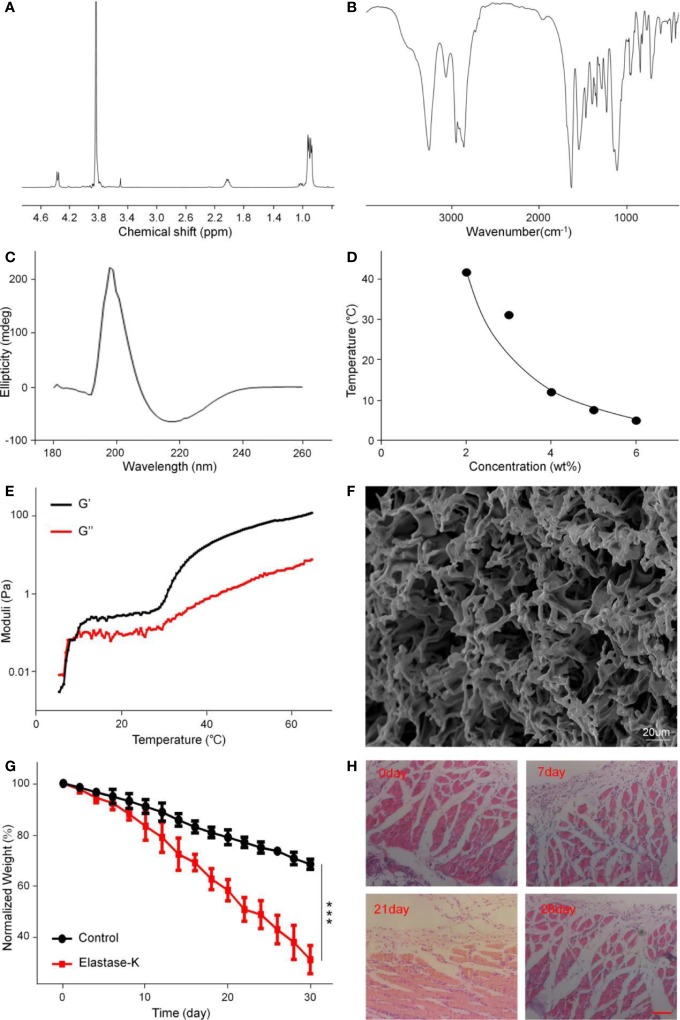
The structural characterization, gelatinization properties, and degradation of mPEG_45_–PLV_19_. **(A)** ^1^H NMR spectra of mPEG_45_–PLV_19_; **(B)** Fourier-transform infrared (FT-IR) spectra of mPEG_45_–PLV_19_; **(C)** circular chromatogram of mPEG_45_–PLV_19_; **(D)** Solution-gel phase diagrams of mPEG_45_–PLV_19_; **(E)** changes of G′ and G″ of mPEG_45_–PLV_19_ in PBS solutions (5 wt. %); **(F)** scanning electron microscope (SEM) image of mPEG_45_–PLV_19_ hydrogels formed at 40; **(G)** mass loss curves of *in vitro* degradation of hydrogels in PBS, and PBS with elastase-K (0.2 mg/ml) groups; **(H)** hematoxylin and eosin staining (H&E) images of the skin tissue near the hydrogels on day 0, 7, 21, and 28, respectively.

mPEG_45_–PLV_19_ obtained by L-Val NCA ROP initiated by mPEG_45_-NH_2_ as a macromolecular initiator (**Figure S1**). **Figure 1A** showed the typical ^1^H NMR spectrum of the block copolymer mPEG_45_–PLV_19_. In the ^1^H NMR spectrum, the peaks at 0.91, 2.04, 3.75–3.89, and 4.36 ppm stood for valine methyl, mPEG terminal methoxy, mPEG backbone, and polymer methine, respectively. It can be seen that mPEG_45_–PLV_19_ was successfully synthesized. The degree of polymerization of PLV was calculated by comparing the integral of the methyl peak of the side chain (-CH_3_) with the methylene peak of polyethylene glycol (-CH_2_CH_2_O-).

The secondary structure of the copolymer was analyzed by FT-IR. From **Figure 1B**, there were characteristic peaks of the amide bonds at 1,548 cm^–1^ and 1,636 cm^–1^, which also showed that copolymers had undergone the main β-sheet conformation.

Circular dichroism is the most widely used instrument for analyzing the secondary structure of proteins. It is a simple, fast, and highly accurate method for studying the conformation of proteins in dilute solutions. As shown in **Figure 1C**, the 0.5 mg/ml polymer solution has a positive absorption characteristic peak at 195 nm and a wavelength range of 180–260 nm. Negative absorption characteristic peak at 226 nm, which is absorption peak specific to the β-sheet conformation, indicating that the block copolymer mPEG_45_–PLV_19_ has undergone the β-sheet conformation

### Phase Diagram, Dynamics, and Morphology Analysis

The phase diagram is derived from the test tube inversion method. The block polymer was dissolved in PBS and a sol-gel transition occurred with increasing temperature. After the test tube is inverted for 30 seconds, the sample does not flow, the temperature is considered to be the phase transition temperature. **Figure 1D** showed the phase diagram of the block copolymer. The gradient concentration of mPEG_45_–PLV_19_ undergoes a sol-gel phase transition under temperature stimulation, and a concentration of 5.0 wt.% is selected as the subsequent experimental gelation concentration. This concentration has fluidity under low temperature conditions, is easy to mix with drugs, is loaded with drugs, and can form a gel upon body temperature stimulation in the body environment, slowly degrades and releases drugs, and has a long-lasting effect on the affected area.

The changes of thermally induced storage modulus (G′) and loss modulus (G″) of mPEG_45_–PLV_19_ were analyzed by rheological experiments. The G′ of mPEG_45_-PLV_19_ increased significantly with temperature and exceeded G″ (**Figure 1E**). When the temperature is below the critical gel temperature (CGTs), G′ is less than G″, the hydrogel was in a liquid state. While the temperature continued to rise above the intersection, G′ exceeds G″, demonstrating the formation of a gel. At the same time, higher G′ also indicates that mPEG_45_-PLV_19_ hydrogel has better mechanical strength The characterization of the drug-loaded gel was shown in **Figure S2**, and the results showed no significant change compared to the unloaded gel.

At the same time, the SEM image of mPEG_45_–PLV_19_ hydrogel is shown in **Figure 1F**. We can observe that the mPEG_45_–PLV_19_ hydrogel forms a 3D network structure with a large number of pores, which indicates that the hydrogel material can load drugs or antibodies are more suitable as carriers and have obvious advantages.

### Degradation Test *In Vivo* and *In Vitro*

The biodegradability of hydrogels is an important factor for clinical application considerations. When the hydrogel is rapidly degraded, the effect of sustained release of the drug cannot be achieved. Conversely, when the hydrogel has poor degradation performance, it may cause side effects such as inflammation at the injection site. The degradation of mPEG_45_-PLV_19_ hydrogel (concentration 5.0%) *in vitro* was analyzed in PBS with elastase K and PBS. As shown in **Figure 1G**, at day 20, the hydrogel mass loss in the elastase K group exceeded 40%, which was significantly higher than 20% in PBS. Degradation was significantly slower in the control group due to lack of enzyme catalysis. The results may indicate that in the absence of elastase K, the mass loss of the gel is only because of the surface erosion of hydrogel. For elastase-K, hydrogel’s polypeptide chain is also rapidly degraded, which accelerates gel loss. Therefore, it can be seen from the degradation rate curve that mPEG_45_-PLV_19_ hydrogel has a more suitable degradation time and is suitable for *in vivo* application. It will not cause adverse reactions due to long stay at the injection site, nor will it cause rapid release of the drug due to too fast degradation.

As shown in **Figure 1H**, *in vivo* degradation experiments can be seen that mPEG_45_-PLV_19_ hydrogel will produce a slight inflammatory response 7 d after implantation, which is mainly because the implantation of the material caused a xenobiotic response of the body. As the hydrogel continued to degrade, the inflammation decreased on the 21st day, and on the 28th day, the tissue section staining was close to normal tissue. Therefore, H&E results show that this hydrogel material will produce a slight inflammatory response in the early stage of implantation, but with the increase of degradation time, the inflammation will gradually disappear without causing serious tissue lesions, indicating that this hydrogel material has good biocompatibility.

### Drug Release *In Vitro* and Local Retention of Hydrogels

**Figure 2:**
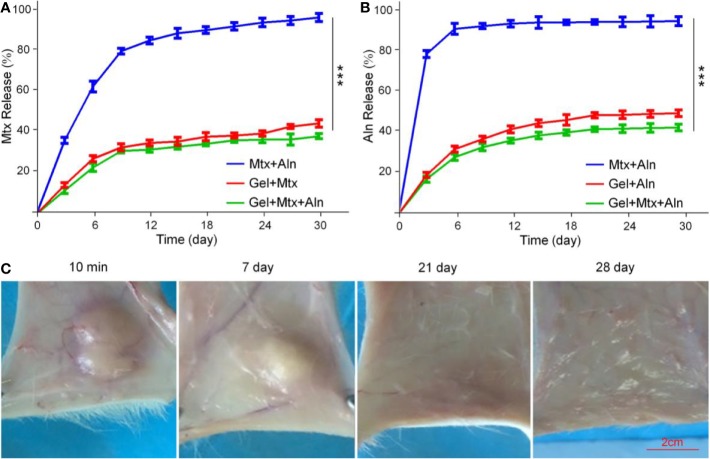
The drugs release behavior *in vitro* and local retention of hydrogels. **(A)** *In vitro* release behavior of Mtx from Mtx+Aln, Gel+Mtx, and Gel+Mtx+Aln; **(B)** *in vitro* release behavior of Aln from Mtx+Aln, Gel+Mtx, and Gel+Mtx+Aln; **(C)** images of localized retention at 10 min, 7, 14, 21, and 28 d after injection of hydrogels. ***P < 0.001.

The *in vitro* release profile of the drug in PBS solution is shown in **Figures 2A, B**. Free Mtx and free Aln showed rapid release behavior, and the release amount reached more than 90% in a short time. However, the sustained release of the drug from the Gel+Mtx+Aln group was significantly different from the cumulative release of the free drug. Therefore, Gel+Mtx+Aln exhibited slowly release of Mtx and Aln, so as to achieve the purpose of sustained and effective drug release at the tumor site.

In **Figure 2C**, the solution rapidly turns into a gel under the influence of body temperature. A small amount of hydrogel was degraded within the first week. The volume of the hydrogel was reduced to below 50% by the second week. After 4 weeks, the hydrogel was not retained locally. This hydrogel retention effect enables sustained local release of the drug.

### Evaluation of Anti-Tumor Effects *In Vivo*

**Figure 3:**
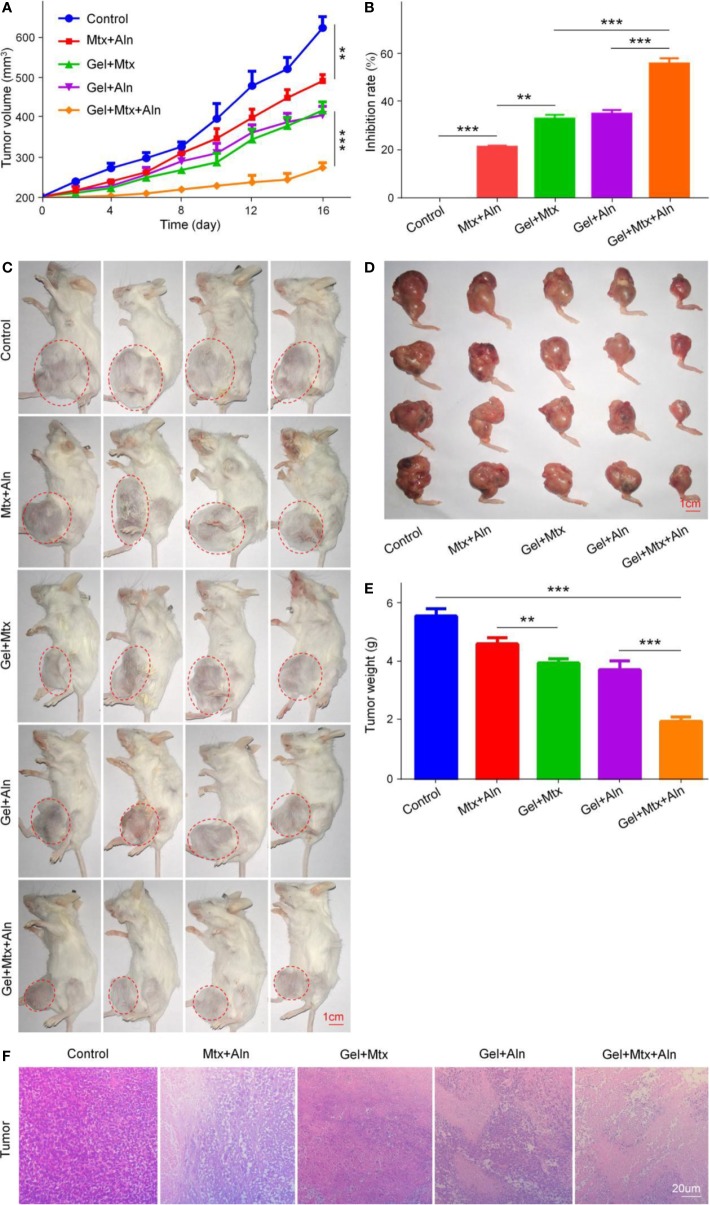
*In vivo* antitumor efficacy of Gel+Mtx+Aln against primary OS. **(A)** Tumor growth curves; **(B)** tumor suppression rate; **(C)** the image of tumor-bearing mice; **(D)** posterior limb tumors. **(E)** Average weight of tibial primary OS tumors. **(F)** Hematoxylin and eosin staining (H&E) of primary tumor. Scale bar = 20 μm (n=4; **P < 0.01, and ***P < 0.001).

Mtx is one of the first-line chemotherapy drugs for OS, which inhibits the synthesis of tumor cells mainly by inhibiting dihydrofolate reductase (Yu et al., 2019; Zhang et al., 2020). Aln is the most important anti-bone resorber for the treatment of bone diseases (Vasikaran, 2001; Rogers et al., 2015). Aln has a high affinity with bone minerals to prevent bone destruction by inhibiting osteoclast activity (Guven et al., 2020). We next evaluated the therapeutic potential of Gel+Mtx+Aln *in vivo*. Treatment was initiated when the tumor in the right leg of the mouse reached 200 mm^3^. In the *in situ* OS mouse model, tumor volume in the PBS group increased significantly over time. As shown in **Figure 3A**, It is worth noting that inhibition of tumor growth was most pronounced by treatment with Gel+Mtx+Aln, however tumor growth was slightly inhibited in other group. As shown in **Figure 3B**, tumor inhibition rates also differed between groups. The inhibition rate of Mtx+Aln was only 21.2%, while the tumor inhibition rates of the gel loaded with Mtx or Aln were 33.0% and 34.8%, respectively. The tumor inhibition rate of Gel+Mtx+Aln group was 55.8%. The same results can be obtained in **Figures 3C, D**. At the end of the experiment, we isolated the right tibia of the mouse and weighed the tumor. The results showed: PBS (5.72 ± 0.24 g) > Mtx+Aln (4.77 ± 0.21 g) > Gel+Mtx (4.11 ± 0.13 g) > Gel+Aln (3.88 ± 0.30 g) > Gel+Mtx+Aln (2.11 ± 0.14 g) (**Figure 3E**), the trend was consistent with tumor volume.

The *in vivo* therapeutic effect of Gel+Mtx+Aln was further evaluated by staining with representative H&E staining of treated mouse K7M2 orthotopic OS tissue. Large and deformed cells with nuclear pyknosis and nuclear fragmentation in the staining results are tumor cells. As shown in **Figure 3F**, in Gel+Mtx+Aln, the tumor necrosis area was smaller than any other group, indicating its enhanced anti-tumor activity.

### Evaluation of Anti-Bone Destruction Effect

**Figure 4:**
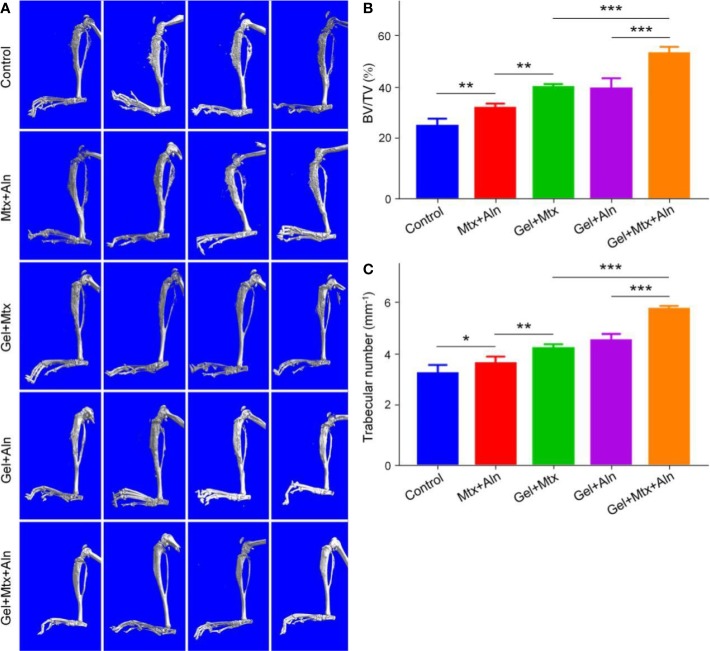
The effect of anti-bone destruction. **(A)** 3D reconstruction of the tibia using micro-CT and **(B**, **C)** semi-quantitative analyses of bone destruction sites (n = 4; *P < 0.05, **P < 0.01, and ***P < 0.001).

Micro-CT is used to observe microstructures due to its unique high resolution. Skeleton is one of the most important applications of micro-CT. To further confirm that Gel+Mtx+Aln has significant tumor suppressive efficacy, the extent of local OS bone destruction is assessed by micro-CT. As shown in **Figure 4A**, in the Gel+Mtx+Aln treatment group, there was no significant bone destruction in the tibia of the mouse, and there was no significant difference compared with the normal mouse tibia. In the PBS group, the surface of the tibia was extremely rough and even defective, and the bone destruction was very serious. Different degrees of bone destruction caused by OS were also clearly seen in the tibia. Micro-CT provides strong support for the remarkable anti-tumor efficacy of Gel+Mtx+Aln. Bone destruction sites in the 3D reconstructed image were set as regions of interest (ROIs), and CTAn software was used to analyze the bone volume/tissue volume (BV/TV) and the number of trabecular bone (Tb. N). The results showed that BV/TV and Tb. N were the highest in the Gel+Mtx+Aln group, indicating that the Gel+Mtx+Aln has a significant anti-bone destruction effect (**Figures 4B, C**).

### Reduced Lung Metastasis of OS

**Figure 5:**
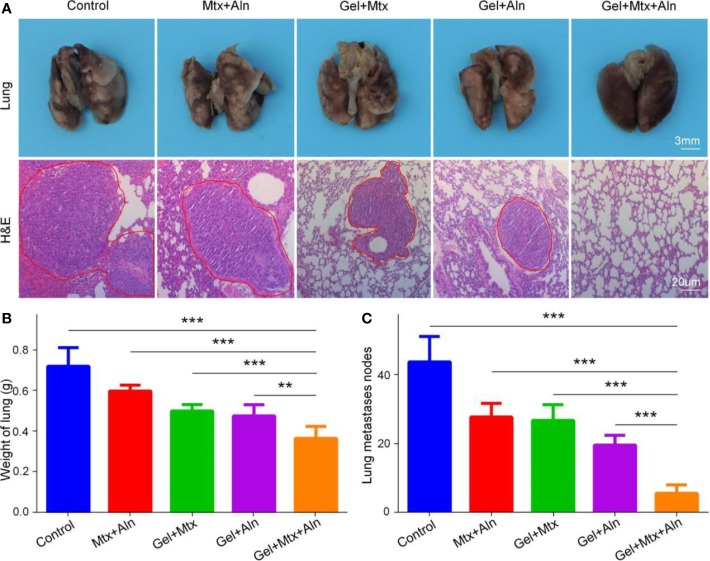
The inhibition of pulmonary metastasis in OS. **(A)** Macroscopic appearance and hematoxylin and eosin staining (H&E) of lung metastatic; **(B)** Lung weight and **(C)** average counted lung metastases. (n = 4; 0.05, **P < 0.01, and ***P < 0.001).

The prognosis of OS has been poor for nearly 30 years. The survival rate at diagnosis without lung metastases and lung metastases varies widely, mainly because of less treatment for OS lung metastasis (Kansara et al., 2014; Friebele et al., 2015). Therefore, effective inhibition of lung metastasis is critical. At the end of the experiment, the lungs of the mice in the different treatment groups were taken out, and after 4 h of fixation with 4% paraformaldehyde, they were evaluated by apparent morphology and H&E staining of the lung sections. As shown in **Figure 5A**, compared with the other groups, the lung metastasis was the smallest in Gel+Mtx+Aln. The lungs were weighed and found in the Gel+Mtx+Aln < Gel+Aln < Gel+Mtx < Gel < PBS group (**Figure 5B**). This is closely related to the number of metastases in the lungs. At the same time, the number of metastases was the highest in the PBS group, but significantly inhibited in the Gel+Mtx+Aln group (**Figure 5C**). This fully demonstrates the efficacy of Gel+Mtx+Aln in inhibiting lung metastasis of OS.

### *In Vivo* Safety Assessment

**Figure 6:**
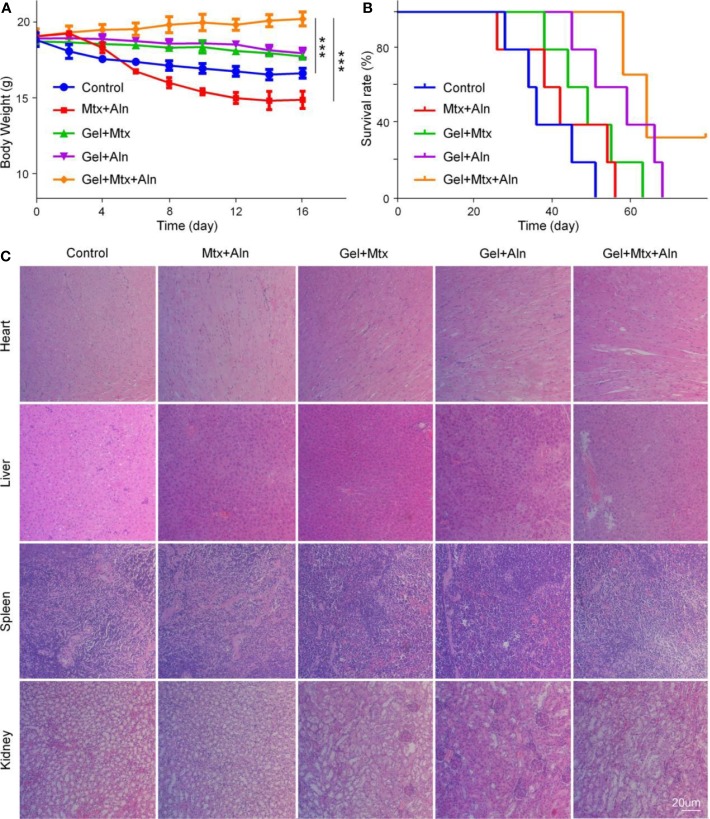
*In vivo* safety. **(A)** Weight changes; **(B)** survival rates; **(C)** mouse organs stained with hematoxylin and eosin staining (H&E). (n = 4; **P < 0.01, and ***P < 0.001).

Studies show that many chemotherapeutic drugs have toxic side effects while treating disease, so their biological safety needs more attention. Here, the safety of the drug is evaluated by monitoring body weight and histopathological analysis of major organs. Weight loss is a comprehensive response to the systemic toxicity of chemotherapy agents. The weight loss of the Mtx+Aln group is significant compared with other groups, which is related to the serious side effects of chemotherapy drugs (**Figure 6A**). However, treatment with Gel+Mtx+Aln did not affect mouse body weight, indicating that the hydrogel improved drug tolerance. Simultaneously monitoring the survival rates of different treatment groups, as shown in **Figure 6B**, the results show that Gel+Mtx+Aln has a significantly longer survival time due to its good antitumor efficacy and higher *in vivo* safety.

Histopathological results showed that Mtx+Aln treatment caused severe renal damage, showing degeneration and necrosis of renal tubular epithelial cells, nucleus pyknosis, and tubular lumen expansion. These signs of toxicity were significantly improved in the Gel+Mtx and Gel+Aln groups. In contrast, mice treated with Gel+Mtx+Aln showed no pathological changes or inflammatory cell infiltration in heart and spleen tissue sections. From **Figure 6C**, it was noted that no significant renal damage was found by Gel+Mtx+Aln, which may be because of the controlled release of the drug *in situ* in the hydrogel.

## Conclusions

This study prepared a degradable, injectable compound delivery system for inhibiting the growth of OS. The drug delivery system consists of a temperature sensitive hydrogel and MTX, ALN. Specifically, the ROP of L-Val NCA elicited by mPEG_45_-NH_2_ produces a polypeptide hydrogel which is then thoroughly mixed by adding Mtx and Aln in the sol state. The obtained sol state is injected into the OS and becomes a gel state at body temperature. Synthetic temperature-sensitive hydrogels have stable mechanical properties, suitable degradation rates and good biocompatibility. Next we studied the advantages of Gel+Mtx+Aln as an OS treatment strategy. The results showed that the drug delivery system had a significant inhibitory effect on K7M2 OS. In summary, the mPEG_45_-PLV_19_ hydrogel designed in this chapter can effectively carry MTX and ALN and may have potential application value in the treatment of OS. At the same time, there were some limitations in the application of mPEG_45_–PLV_19_. The preparation of mPEG_45_-PLV_19_ hydrogel was complicated and needs further optimization. In addition, the specific injection site and dose of the hydrogel for clinical treatment remain to be explored.

## Data Availability Statement

All datasets generated for this study are included in the article/**Supplementary Material**.

## Ethics Statement

The animal study was reviewed and approved by the Animal Care and Use Committee at Jilin University.

## Author Contributions

KL, JY, and MP proposed and designed the experiments. HS, KL, and CC carried out the experiments with the help of QT and XW. HS and KL drafted the manuscript and interpreted the data. KL, DZ, JY, and MP revised the manuscript.

## Conflict of Interest

The authors declare that the research was conducted in the absence of any commercial or financial relationships that could be construed as a potential conflict of interest.
