# Supplementary material for: Locally Controlled Release of Methotrexate and Alendronate by Thermo-Sensitive Hydrogels for Synergistic Inhibition of Osteosarcoma Progression
Source: Front Pharmacol. 2020 May 19;11:573. doi: 10.3389/fphar.2020.00573 (PMC7248331; doi:10.3389/fphar.2020.00573)
Supplement: Supplementary file 1 [file DataSheet_1.docx]

Supporting Information

Locally Controlled Release of Methotrexate and Alendronate by Thermo-sensitive Hydrogels for Synergistic Inhibition of Osteosarcoma Progression

*Hongli Shan^1^, Ke Li^2^, Duoyi Zhao^2^, Changliang Chi^3^, Qinyuan Tan^3^, Xiaoqing Wang^3^, Jinhai Yu^4^* and Meihua Piao^5^**

*^1^ Department of Clinical Laboratory, The First Hospital of Jilin University, Changchun 130021, P. R. China, ^2^ Department of Orthopedics, the Fourth Affiliated Hospital of China Medical University, Shenyang 110032, P. R. China, ^3^ Department of Urology, the First Hospital of Jilin University, Changchun 130021, P. R. China, ^4^ Department of Gastrointestinal Surgery, The First Hospital of Jilin University, Changchun 130021, P. R. China, ^5^ Department of Anesthesiology, The First Hospital of Jilin University, Changchun 130021, P.R. China*

*Correspondence:

Meihua Piao

pumh@jlu.edu.cn

Jinhai Yu

jidayujinhai@126.com

**MATERIALS AND METHODS**

**Characterizations**

The composition of the PLV hydrogel was determined by proton nuclear magnetic resonance (400 MHz NMR, Billerica, MA, USA; ^1^H NMR) spectroscopy. Fourier transform infrared spectroscopy was obtained by potassium bromide compression method using Fourier transform infrared spectroscopy (Bio-Red Win-IR, MA, USA; FTIR). The secondary structure of the PLV hydrogel was determined by a circular dichroism (JASCO J-810, Japan; CD) at a concentration of 0.5 mg/mL, a wavelength range of 180−260 nm, and the number of repeated scans was 3 times. The input is 1 nm, and the sampling time of a single data is 0.5 s.

**Phase Diagram, Dynamics and Morphology Analysis**

The phase diagram was obtained by inverting the test tube, and the block copolymer was dissolved in a phosphate buffer solution (PBS) to a solution having a concentration of 2.0-6.0 wt%, and after stirring at 4 °C overnight, 200 μL of the solution was taken with a pipette. The liquid was placed in a 2.0 mL glass vial, two parallel samples were taken at each concentration, the temperature was raised from 5 °C, 2 °C was raised every 10 minutes, raised to 65 °C, and the glass bottle was tilted. If the liquid surface does not flow, it indicates that the copolymer has undergone a phase transition, from a sol state to a gel state, and the temperature at which each phase undergoes a phase transition is recorded and plotted.

The dynamic viscoelastic properties of the PLV hydrogel were measured by using a US302 rheometer (Anton Paar). The mPEG_45_-PLV_19_ solution (5.0% wt%) was placed between the parallel plates (the diameter was set to 25.0 mm and the gap was set to 0.5 mm). The outer edge of the sandwich sample was sealed with a thin layer of silicone oil to prevent evaporation of moisture. Data were collected at a controlled strain gamma of 1% and a frequency of 1 rad/s at a heating rate of 0.5 °C/min. Secondly, the morphology of the hydrogel was analyzed by scanning electron microscopy (XL 30 ESEM FEG; SEM).

**Biodegradation *in vitro* and *in vivo***

The copolymer solution (500 ul, 5% wt) was added to a 3.0 mL glass vial and incubated at constant temperature (37 °C) for 10 minutes to obtain an mPEG_45_-PLV_19_ hydrogel. A PBS buffer (pH 7.4) containing 0.2 mg/mL elastase K was used as a degradation medium, and a hydrogel incubated only in PBS buffer was used as a control. A buffer solution (2.0 mL) was added to the top of the hydrogel at 37 °C, the culture medium was changed once a day, and the remaining gel was accurately weighed every two days to monitor the rate of gel degradation and plotted as a graph, each sample is set up in two parallel samples.

500 uL of a 5.0 wt% copolymer solution was injected into the subcutaneous part of the back of the rat, and the rats were sacrificed at 0, 7, 21, and 28 days, respectively, and the subcutaneous tissue at the back gel was photographed. After paraformaldehyde was fixed, it was embedded in paraffin, cut into pieces for H&E staining, and then photographed by a fluorescence inverted microscope (Nikon TE2000-U, Japan).

**Animal and Cell**

Osteosarcoma (OS) cell line K7M2 was cultured in DMEM medium (Invitrogen) to which 10% newborn calf serum (Beijing Solabao Technology Co., Ltd.) was added. Ensure cell viability is >95% prior to experimentation.

BALB/c female mice (4 weeks) were purchased from Beijing Weitong Lihua Experimental Animal Technology Co., Ltd. for *in vivo* anti-tumor experiments of PLV hydrogels. All mice were housed in a standard specific pathogen free environment with a control temperature of 20−22 °C and a light dark cycle of 12 h. All mice were treated humanely and treated according to the protocol approved by the Animal Protection Agency and the Use Committee of China Medical University.

**Histopathological Analysis**

At the end of the experiment, the tumor and main organs (*i.e.*, the heart, liver, spleen, lung and kidney) of the mice were isolated, fixed in 4% (*W*/*V*) PBS buffered paraformaldehyde overnight, and then embedded in paraffin. Paraffin-embedded tissues were cut into sections for H&E staining. Histopathology was examined by microscope (Nikon Eclipse Ti, Ardmore, PA).

**Statistical Analysis**

All data are expressed as mean ± standard deviation (SD). Student's t-test was used to verify differences between the different groups. **P* < 0.05 indicates statistical significance, ***P* < 0.01 and ****P* < 0.001 indicate significant statistical significance.


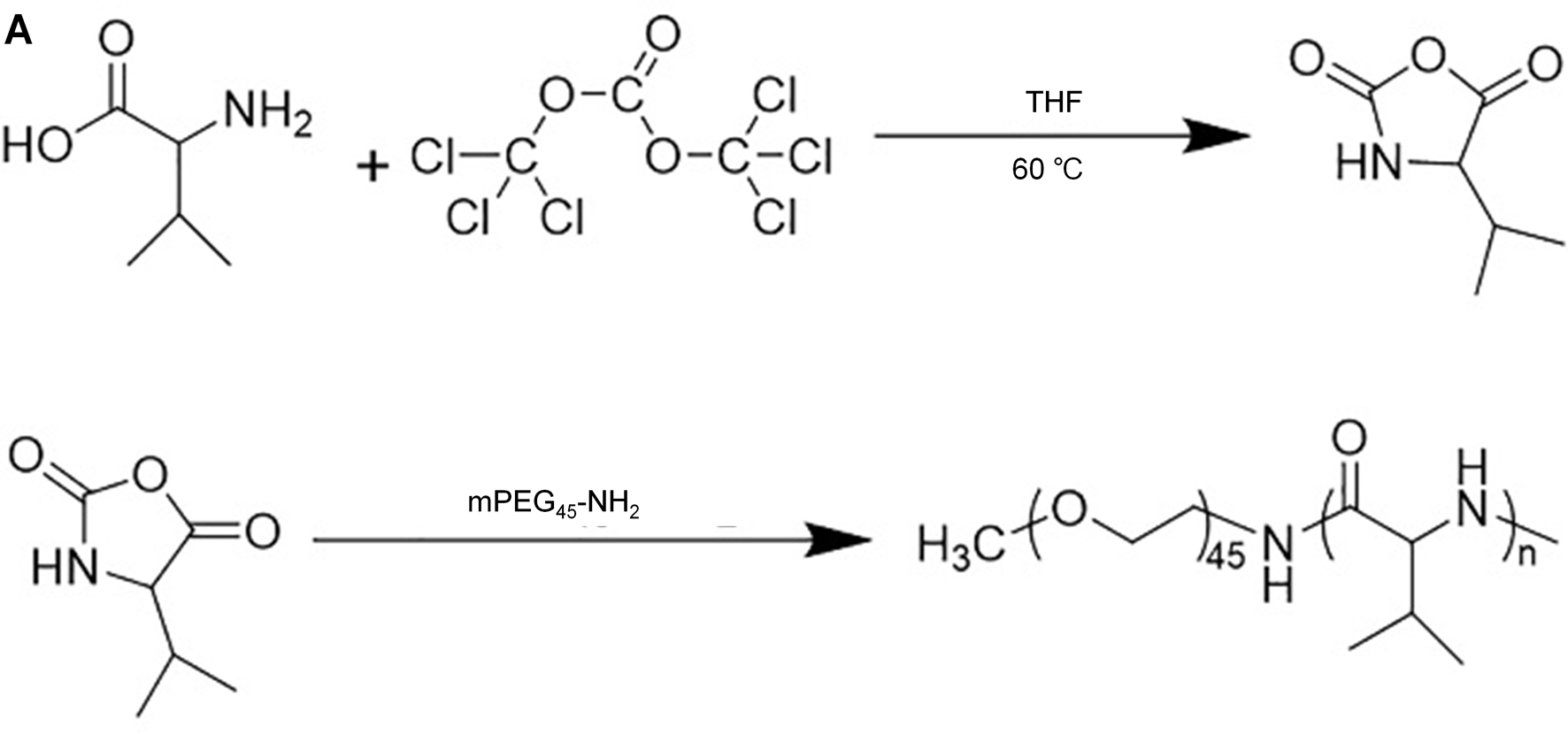


**FIGURE S1.** mPEG_45_-NH_2_ initiation of Val NCA ring-opening polymerization to prepare mPEG45–PLV.


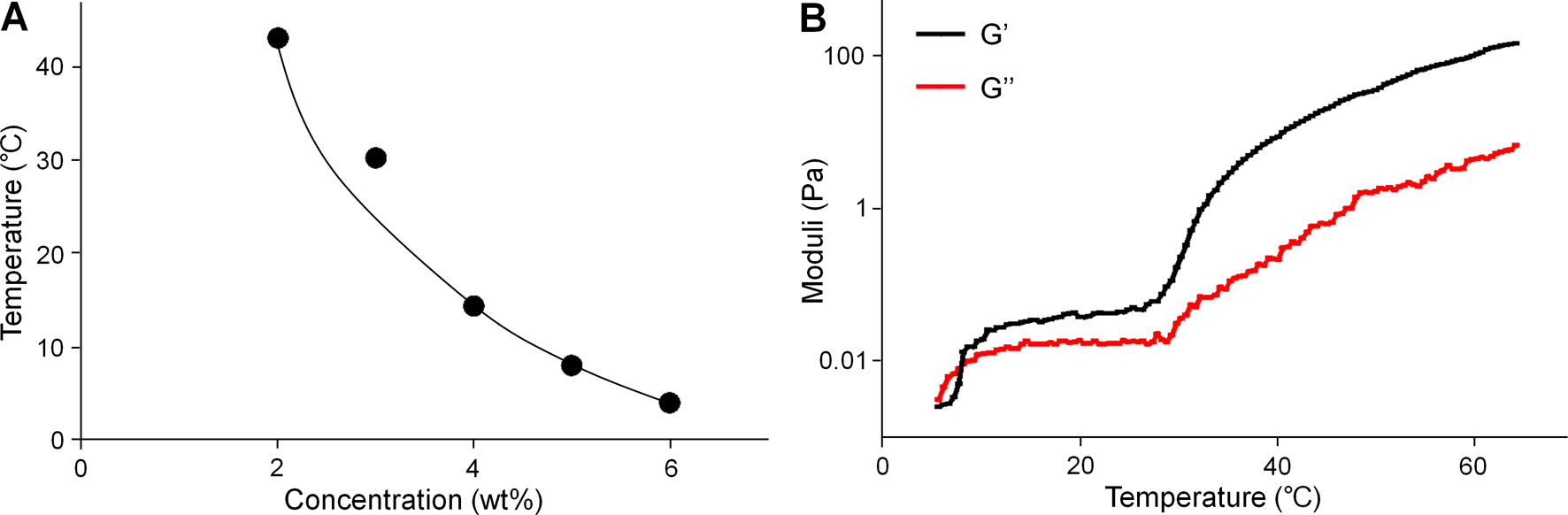


**FIGURE S2.** The characterization of drug-loaded gel. (A) Solution-gel phase diagrams; (B) Changes of G' and G'' of drug-loaded gel in PBS solutions (5 wt. %).
